# An integrated approach in a case of facioscapulohumeral dystrophy

**DOI:** 10.1186/1471-2474-15-155

**Published:** 2014-05-15

**Authors:** Stefano Pasotti, Bruno Magnani, Emanuela Longa, Giuseppe Giovanetti, Albino Rossi, Angela Berardinelli, Rossella Tupler, Giuseppe D’Antona

**Affiliations:** 1 LUSAMMR Laboratory for Motor Activities in Rare Diseases, Voghera, University of Pavia, Via Forlanini 6, Pavia 27100, Italy; 2 Department of Public Health, Human Anatomy, University of Pavia, Via Forlanini 6, Pavia 27100, Italy; 3 Department of Child Neuropsychiatry, C. Mondino Institute, Pavia, Italy; 4 Department of Biomedical Sciences, University of Modena and Reggio Emilia, Modena, Italy; 5 Department of Molecular Medicine, and LUSAMMR Laboratory for Motor Activities in Rare Diseases, Voghera, University of Pavia, Via Forlanini 6, Pavia 27100, Italy

**Keywords:** Muscular dystrophies, Exercise therapy, Dietary supplements, Creatine, Amino acids

## Abstract

**Background:**

Muscle fatigue, weakness and atrophy are basilar clinical features that accompany facioscapulohumeral dystrophy (FSHD) the third most common muscular dystrophy.

No therapy is available for FSHD.

**Case presentation:**

We describe the effects of 6mo exercise therapy and nutritional supplementation in a 43-year-old woman severely affected by FSHD.

**Conclusion:**

A mixed exercise program combined with nutritional supplementation can be safely used with beneficial effects in selected patients with FSHD.

## Background

Selective muscle atrophy with weakness and early fatigue [1] are main features of facioscapulohumeral dystrophy (FSHD), the third most common muscular dystrophy (MD).

No therapy is available for FSHD. Nevertheless, patients usually report some improvement related to physical exercise and the use of nutritional supplements (NS). These effects are not currently substantiated by appropriate investigations. In particular the effect of exercise training has not been fully examined mainly due to concerns that it may trigger disease progression. However recent observations suggest that in FSHD post-exercise creatine kinase (CK) elevation (indicator of muscle damage) normalizes within one day compared with the pre-exercise values [2].

Again, the role of NS in MD remains to be elucidated. Recent observations suggest that among supplements creatine and Branched Chain Amino Acids (BCAA) may exert positive effects as they may act by limiting exercise-induced injury [3]. The positive changes due to creatine appear to be safely enhanced by coadministration of conjugated linoleic acid (CLA) [4].

The aim of this case report is to show the effects of a controlled exercise training and NS in a FSHD patient.

## Case presentation

A 43-year-old wheelchair-bound woman from 2006, affected by FSHD, underwent 6-month exercise therapy associated with NS to study metabolic and possible changes in motor function related to the program.

The patient was born from uneventful pregnancy and delivery to non-consanguineous parents, with family history negative for MD.

The patient carries a “de novo” D4Z4 reduced allele with 2 repeats (14 kb).

When she was 2 yrs her parents noted difficulty of walking and waddling gait. At 4 she was diagnosed having a muscle disease. At 11 she was clinically diagnosed with a progressive MD. At 30 neurological examination revealed left steppage gait, facial weakness, atrophy of shoulder girdle muscles with bilateral scapular winging, atrophy of bilateral pectoralis, infraspinatus and supraspinatus muscles, moderate biceps brachii and severe deltoid weakness, foot extensor weakness and proximal hip weakness, lumbar hyperlordosis; lower extremities tendon reflexes were absent, sensory examination was normal. In 1982 a muscle biopsy revealed myopathic changes compatible with FSHD.

Presently she presents severe muscle hypotrophy and proximal-distal weakness leading to loss of ambulation in 2006 and severe lumbar hyperlordosis (FSHD score 12 according to Lamperti et al. [5]).

**Figure 1:**
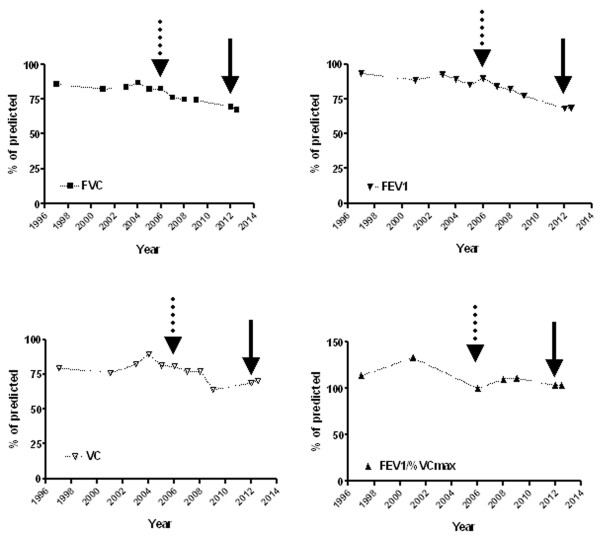
**Temporal change (from 1997 to 2012) of selected pulmonary function tests expressed as percentage of predicted (FVC, upper left; VC, lower left; FEV1, upper right; FEV1/VCmax, lower right) showing the progression from mild to moderate restrictive respiratory failure in 43y old FSHD patient.** Dotted arrow indicates the year of bound to wheelchair; solid arrow indicates the start of sport therapy protocol.

In 2001 respiratory function assessment first revealed mild respiratory restrictive insufficiency (%pred: VC 75.9; FVC 82.3; FEV1 88.50; FEV1%/VCmax 133, see Figure 1).

## Methods

The patient (180 cm, 71.4 Kg) participated in the study after providing written informed consent. Procedures were conducted according to the Declaration of Helsinki. The protocol had a duration of 6 mo, 4 days/week. Before and after exercise and nutrition intervention the following evaluations were performed:

### Oxygen consumption

Considering that skeletal muscle metabolism is the major determinant of resting energy expenditure (REE), we explored the impact of disease and training/supplementation on REE by indirect calorimetry (Quark *b*^*2*^, Cosmed) in supine position (12 h fasting at 9.00 a.m.).

Endurance performance was evaluated with incremental stepwise ramp test (ISRT) on a handbike (Monark Trainer 881E). After three minutes at rest the subject exercised at 0 Watt as warm-up. Then the workload was increased by 2.5 Watt/step with a step duration of 2.5-min until exhaustion (cadence 60 rpm). Gas flows were continuously recorded breath-by-breath using the Quark *b*^*2*^. Beat-by-beat heart rate (Polar RS400) and ECG were monitored. Breath-by-breath data were averaged per 10s at the end of each workload. Peak cardiorespiratory responses were defined as the maximal averaged values registered over 10s during the last test stage [6]. Peak oxygen uptake (VO_2peak_), minute ventilation (VE), and Borg score at 7.5 Watt are reported in Table 1.

**Table 1:** Body composition, blood and functional parameters before and intervention

**Parameter**	**Before**		**After**		**UM**	**n.v.**
***Body composition***						
Body mass	71.4		62		Kg	
BMI	21.9 ± 0.1		19.2 ± 0.1		%	18.5-24.9
TBW	33.8 ± 0.2		38.2 ± 0.3		%	55-62
ECW	50.2 ± 0.3		53.1 ± 0.1		%	40% TBW
ICW	50.3 ± 0.4		47.1 ± 0.2		%	60% TBW
FFM	64.2 ± 0.3		72.1 ± 0.1		%	Min 75
FM	36.2 ± 0.2		28.1 ± 0.1		%	Max 25
***Blood parameters***						
HDL	42		46		mg/dc	>43
Calcium	8.3		8.9		mg/dc	8.5-11
Total Proteins	5.9		6.6		g/dc	6-8
Albumin	52.5		56.6		%	55.8-66.1
Alpha1 globulin	5.5		4		%	2.9-4.9
Alpha2 globulin	12.7		11		%	7.1-11.8
Beta1 globulin	6.3		5.5		%	4.7-7.2
Beta2 globulin	4.9		4.4		%	3.2-6.5
Gamma globulin	18.1		18.5		%	11.1-18.8
A/G	1.11		1.3		-	1.26-1.95
***Functional parameters***						
HR	68		65		b/min	
HRpeak	143		141		b/min	
REE	1358		1332		ml · min^−1^	
VO_2(7.5Watt)_	11.76		7.9		ml/Kg/min^−1^	
VE	30		20		L/min	
Borg Scale_7.5Watt_	17		14			
Muscle district	Right	Left	Right	Left		
Shoulder abductors	16,7*	21,6†	38.2*	39.2†	N	
Elbow flexors	25,5‡	16,7‡	20,6‡	23,5‡	N	
Elbow extensor	6,9‡	12,7‡	11,7‡	10,7‡	N	

BMI: body mass index; TBW: total body water; ECW: extracellular water; ICW: intracellular water; FFM: fat free mass; FM: fat mass; HDL: high density lipoprotein; A/G: albumin globulin ratio. HR: heart rate; mHR: maximal heart rate (mHR = 206.9 – (0.67*age); REE: resting energy expenditure; VO_2(7.5Watt)_: oxygen consumption at 7.5 Watt load; VE: ventilation exchange; Isometric force in Newton; *23°; † 28°; ‡ 90°.

### Body composition

Whole body bioelectrical impedance analysis (BIA) was made with a single frequency (50 kHz) analyzer (BIA-101, RJL/Akern). Test-retest reliability was assessed in two separate measurements 2 h apart. Means and standard deviations for all variables are reported in Table 1.

### Isometric strength

Isometric strength (maximal voluntary contraction, MVC) was quantified by means of a dynamometer (Muscle Lab 4000E). The patient carried MVC of the following groups: elbow flexors, elbow extensors, shoulder abductors. Each trial lasted 3 s and the rest time was of 30s and the mean peak force (Nm) was calculated.

### Spirometry

Pulmonary function tests (PFTs) were recorded by using Cosmed Pony spirometer. The PFTs recorded before and after training are reported in Table 1. Temporal change (from 1997 to 2012) of selected PFTs (FVC, VC, FEV1, FEV1/VCmax) is described in Figure 1.

### Blood analysis

Routine blood and urine analysis were performed before and after treatment. Blood parameters changed after intervention are reported in Table 1. No change in CK levels was observed after training (initial value 87 U/L; final value 86 U/L (n.v. 15–150).

### Training

The patient underwent a mixed endurance/strength training. Heart rate was monitored during endurance sessions. The following protocol was adopted (2 time/wk endurance, 2 time/wk strength). Endurance training on assisted arm cycle ergometer (warm up, HR 90 min; work, 4 kCal/min or HR 130 min^−1^): wk 1, 5 min warm up, 10 min work; wk 2–12, 5 min warm up, 15 min work; wk 13–24, 5 min warm up, 20 min work. Strength training with green Thera-band®: 8 repetition 2 series, 5 min rest between series, for each muscular district. To obtain a load of 50-60% of MVC the green Thera-band® was stretched of a defined% of the initial length: for lateral abduction the patient was instructed to stretch the Thera-band® of about 50% of the band initial length, for biceps flexion and triceps extension of about 25%. The sessions were performed at patient’s home without supervision. Periodical Skype® meetings were organized to verify the correct execution of training.

Respiratory muscle training consisted in 4 time/wk-2 min sessions (50% vital capacity at 20 min^−1^) of isocapnic hyperpnea. Training was carried out with a device (Spirotiger®) that operates on rebreathing principle [7]. Before training subject was instructed to keep the tidal volume at 50% of the vital capacity and to follow the device metronome for frequency pacing.

### Nutritional supplementation

Nutritional supplementation included BCAA enriched mixture, creatine plus CLA.

The amino acid supplement consisted in a BCAA-enriched formula with 2:1:1 leucine:isoleucine:valine ratio (BigOne, Professional Dietetics), and already known to exert beneficial effects on skeletal muscle [8-10] (0.1 gr/kg/day in two doses at breakfast and dinner). Creatine monohydrate (CreaATP, Syform, 0.1gr/day) was administered in combination with CLA (Syform, 2.4 gr/day corresponding to 3 pearls/day at breakfast or 2 h before strength exercise) [11].

## Results

At the end of the protocol we observed an improvement of the patient’s nutritional status as demonstrated by significant improvement of anthropometric (BMI, FFM) and biochemical (serum proteins) parameters. In particular a reduction of body mass (−13.17%) associated with reduced fat mass (FM, −8%) and improved fat free mass (FFM +8%) was found (Table 1). Indeed beneficial changes in biochemical blood parameters included increased total protein concentration, albumin percentage, and albumin/globulin ratio which were reported to normal values (Table 1). Functionally a modest increase of peak force was observed only in shoulder abductors bilaterally whereas no change was observed in elbow flexors and extensors (Table 1). An amelioration of peak oxygen consumption (VO_2peak_), ventilation exchange, and an improvement of Borg score during submaximal exercise suggested the appearance of a certain degree of muscular adaptation to training and possibly an amelioration of the oxidative metabolism (Table 1). This was demonstrated by increased time/power to exhaustion during incremental test. Finally after intervention substantial steadiness of several PFTs in comparison with initial evaluation (FVC before: 69.6, after 67.4; VC: 68.7 before, 70.4 after; FEV1 before 68.2; after 68.5; FEV1/FVC% before 104.9, after 108.8) was observed (Figure 1).

## Conclusions

Results from our report suggest that a specific exercise therapy and supplementation program can be safely used in selected FSHD patients but the relative contribution of the adopted interventions, exercise and dietary supplementation, and whether their synergy may contribute to the observed improvements remains unknown and deserves future controlled studies.

Physical exercise may represent one possible way to counteract atrophy and fatigue in FSHD but its effects have not been fully investigated due to concerns that it may ultimately trigger disease progression. Recent meta analysis suggests that moderate-intensity strength training does not appear to harm individuals with FSHD, but there is insufficient evidence that it provides benefits [12]. However initial observations have shown that at least aerobic training is beneficial [13].

The concept that NS might have effects in prevention or treatment of MD is experiencing a new revival [11]. However the lack of unequivocal results and flaws in the choice of supplements and protocols to be adopted leads to an assumption mostly based on empirical attitude. Among supplements creatine and BCAA may exert positive effects as they act by limiting load-induced muscle injury. In particular creatine improves muscle mass and performance in combination with strength training [14] and attenuates exercise-induced alteration of markers of cell death/lysis [15] whereas BCAA may act by maximizing protein synthesis [4]. Actually, the only creatine trial in MD, including FSHD, showed a small but significant improvement of 3% in muscle strength and 10% in daily life activities [16]. No clinical BCAA trials are currently available.

These very preliminary indications suggest positive effects of training and selected supplements in muscle disease [4], therefore we applied an integrated approach based on endurance/strength and respiratory training combined with NS with BCAA and creatine + CLA for six months.

In this context, here we compared the results of this approach on physical capacity and blood profile of a 43y old wheelchair bound FSHD patient with severe proximal and distal muscular impairment, and moderate restrictive respiratory insufficiency.

In particular, our study revealed an improvement of blood protein profile, body mass and composition, and an amelioration of peak oxygen consumption whereas no change in resting energy expenditure was observed. Furthermore after initiation of the exercise nutrition intervention, several PFTs demonstrated stable values. Importantly, the program was well tolerated by the patient as no muscle soreness, change in CK serum levels or intolerance to supplements were observed.

### Limitations of the study

Our study has limitations. The design of the study was not scientifically sound to answer the questions of which is the relative contribution of the adopted interventions to the observed improvements and whether the obtained results can be reproducible in all FSHD patients or in selected subgroups. Indeed we cannot rule out whether a possible contribution to the observed changes in body mass and composition derives by concomitant self-determined changes in diet lifestyle. Moreover, specific outcome measures relevant to daily life should be introduced in the study. Notwithstanding these limitations, the results shown in this paper are novel and may be helpful to encourage scientists and clinicians to plan more research in this field.

## Consent

The participant signed a consent approved by which informed the participant that the data would be published in medical journals without personally identifiable information. A copy of the signed informed consent is available for review upon request.

## Abbreviations

FSHD: Facioscapulohumeral dystrophy; MD: Muscular dystrophy; NS: Nutritional supplements; VO2peak: Peak oxygen consumption; VE: Ventilation exchange; REE: Resting energy expenditure; MVC: Maximal voluntary contraction; BIA: Bioelectrical impedance analysis; CK: Creatine kinase; BCAA: Branched chain amino acids; CLA: Conjugated linoleic acid; PFTs: Pulmonary function tests; VC: Vital capacity; FVC: Forced vital capacity; FEV1: Forced expiratory volume at the first second; ISRT: Incremental stepwise ramp test; FM: Fat mass; FFM: Fat free mass; BMI: Body mass index; TBW: Total body water; ECW: Extracellular water; ICW: Intracellular water; HDL: High density lipoprotein; A/G: Albumin globulin ratio; HR: Heart rate.

## Competing interests

Publication of this study was funded by the manufacturer of BigOne (Professional Dietetics, Milano). The authors of this paper have no financial relationship with Professional Dietetics or with Syform nutritional supplements.

## Authors’ contributions

SP, BM, EL, GG, AR participated in data collection. AB, RT participated to the data interpretation and manuscript preparation. GD served as the principal investigator and contributed to study design, data collection and interpretation, and manuscript preparation. All authors read and approved the final manuscript.

## Pre-publication history

The pre-publication history for this paper can be accessed here:

http://www.biomedcentral.com/1471-2474/15/155/prepub
